# Behavior-associated and post-consumption glucose entry into the nucleus accumbens extracellular space during glucose free-drinking in trained rats

**DOI:** 10.3389/fnbeh.2015.00173

**Published:** 2015-07-02

**Authors:** Ken T. Wakabayashi, Eugene A. Kiyatkin

**Affiliations:** In-Vivo Electrophysiology Unit, Behavioral Neuroscience Branch, National Institute on Drug Abuse—Intramural Research Program, National Institutes of HealthBaltimore, MD, USA

**Keywords:** glucose, drinking behavior, high-speed amperometry, enzyme-based glucose sensors, metabolism, cerebral blood flow, neuronal activity

## Abstract

Glucose is the primary energetic substrate for the metabolic activity of brain cells and its proper delivery from the arterial blood is essential for neural activity and normal brain functions. Glucose is also a unique natural reinforcer, supporting glucose-drinking behavior without food or water deprivation. While it is known that glucose enters brain tissue via gradient-dependent facilitated diffusion, it remains unclear how glucose levels are changed during natural behavior and whether the direct central action of ingested glucose can be involved in regulating glucose-drinking behavior. Here, we used glucose biosensors with high-speed amperometry to examine the pattern of phasic and tonic changes in extracellular glucose in the nucleus accumbens (NAc) during unrestricted glucose-drinking in well-trained rats. We found that the drinking behavior is highly cyclic and is associated with relatively large and prolonged increases in extracellular glucose levels. These increases had two distinct components: a highly phasic but relatively small behavior-related rise and a larger tonic elevation that results from the arrival of consumed glucose into the brain’s extracellular space. The large post-ingestion increases in NAc glucose began minutes after the cessation of drinking and were consistently associated with periods of non-drinking, suggesting that the central action of ingested glucose could inhibit drinking behavior by inducing a pause in activity between repeated drinking bouts. Finally, the difference in NAc glucose responses found between active, behavior-mediated and passive glucose delivery via an intra-gastric catheter confirms that motivated behavior is also associated with metabolic glucose use by brain cells.

## Introduction

Glucose is the main energetic substrate for the metabolic activity of brain cells (Siesjo, 1978; Sokoloff, 1999; Mergenthaler et al., 2013) and its proper delivery to the brain is essential for maintaining normal neural activity and functions. Unlike most neurochemicals that are locally synthetized and released either in synapse or into the extracellular space due to neural activation, glucose enters the brain tissue from the arterial blood via gradient-dependent facilitated diffusion via the glucose transporter (GLUT-1; Duelli and Kuschinsky, 2001). Rapid, but relatively modest entry occurs via increases in local blood flow due to proximal neural activation (Fellows et al., 1992; Silver and Erecińska, 1994; Attwell et al., 2010), while slower but larger elevations result from a global rise in glucose blood levels (de Vries et al., 2003; Dunn-Meynell et al., 2009; Dash et al., 2013).

Glucose is also a unique natural reinforcer, supporting consummatory behavior independently of food or water deprivation. While much attention has been focused on the taste of glucose as its primary reinforcing action (Sclafani, 1987; De Araujo, 2011), glucose readily enters the brain after its consumption and interacts with multiple neuronal glucoreceptors, inducing central neural effects at a behaviorally relevant time-scale. While glucoreceptors have been identified in electrophysiological studies on neural cells involved in regulating the organism’s activity state and motivational processes (Routh, 2002; Levin et al., 2002; Burdakov et al., 2005; Routh et al., 2014; Sakurai, 2014; Sheng et al., 2014), due to technical limitations in glucose detection in awake animals it remains unclear whether these receptors are activated during natural glucose ingestion and whether a direct central action of glucose occurs rapidly and strongly enough to be involved in regulating glucose-drinking behavior.

In this study, we used glucose biosensors coupled with high-speed amperometry to examine the pattern of phasic and tonic changes in extracellular glucose during unrestricted glucose-drinking behavior in well-trained rats. We used a naturalistic model, where extensively trained rats consumed a freely available 10% glucose solution without any operant actions or experimenter-imposed restrictions. Our recordings were conducted in the nucleus accumbens (NAc), a critical brain structure involved in motivation and reinforcement (Wise and Bozarth, 1987; Di Chiara, 2002). This area contains a high density of GLUT-1 transporters (Zeller et al., 1997), has a high level of glucose utilization (Gonzalez-Lima et al., 1993), and exhibits highly dynamic changes in extracellular glucose in response to naturally arousing stimuli (Kiyatkin and Lenoir, 2012; Kiyatkin and Wakabayashi, 2015) and during limited-access glucose consumption in minimally trained rats (Wakabayashi et al., 2015). To differentiate the behavioral and post-consumption contributions to changes in glucose concentration, a subgroup of rats were implanted with a chronic intra-gastric catheter, allowing us to examine how NAc glucose levels are affected by passive glucose administration at the volumes naturally consumed in the behavioral context.

The present study is an extension of our previous work, where we examined the fluctuations in NAc glutamate and glucose in moderately trained rats while drinking a predetermined, limited quantity (5 ml) of glucose (Wakabayashi et al., 2015). While the present study confirmed several previous conclusions, the current study has important differences in experimental design. Specifically, the paradigm of free, unrestricting glucose drinking in highly trained rats used here allows us to reveal the differences between cue-initiated and self-initiated drinking and address new questions focused on the relationship between post-ingestion glucose elevations and the pattern of drinking behavior.

## Materials and Methods

### Subjects

Data from seven adult male Long-Evans rats (Charles River Laboratories, Raleigh, NC, USA) weighing 660 ± 60 g at the time of surgery were used in this study. Rats were housed individually in a climate controlled vivarium maintained on a 12–12 h light-dark cycle (lights on at 8:00 AM), with food and water available ad libitum. All experiments complied with the “Guide for the Care and Use of Laboratory Animals” (8th edition, 2011, US National Research Council) and experimental protocols were approved by the Intramural Research Program (NIDA) Animal Care and Use Committee.

### Pre-Training

Rats were extensively trained with an unlimited volume of 10% glucose solution presented in a glass bottle for 4 h per a 6-h session for 7 days during a 1-month period (once a week for first 3 weeks and three times a week for the last week). Training occurred inside a Plexiglas chamber (42 × 42 × 30 cm), with a monitoring of consumption volume every 4 min during the entire period of access.

### Surgeries

Surgical procedures for electrochemical experiments have been described in detail elsewhere (Wakabayashi and Kiyatkin, 2012, 2015). Briefly, under general anesthesia, each rat was unilaterally implanted with a BASi guide cannula (Bioanalytical Systems, West Lafayette, IN, USA) for later insertion of the electrochemical sensor in the NAc shell, a critical brain structure involved in sensorimotor integration, behavioral regulation and reward (Wise and Bozarth, 1987; Di Chiara, 2002). Target coordinates were: AP +1.2 mm, ML ±0.8 mm, and DV +7.6 mm from the skull surface, according to coordinates of Paxinos and Watson (1998). The cannula was secured with dental acrylic in a head mount anchored to the skull.

Three rats were also equipped with a chronic intra-gastric catheter during the same surgery. The catheter was implanted into the forestomach (the upper area with minimal vascularization) and secured to the stomach wall. After closure of the abdominal wall, the catheter was fed subcutaneously to an injection port on the head mount. During recovery, the catheter was flushed daily with water to maintain patency.

### Electrochemical Sensors

Commercially produced glucose oxidase-based biosensors (Pinnacle Technology, Inc., Lawrence, KS, USA) coupled with fixed-potential amperometry have been extensively used in our previous studies (Kiyatkin and Lenoir, 2012; Kiyatkin et al., 2013; Wakabayashi and Kiyatkin, 2014, 2015; Kiyatkin and Wakabayashi, 2015; Wakabayashi et al., 2015). These reports describe in detail multiple issues regarding the sensitivity/selectivity of these sensors, their *in vitro* and *in vivo* performance, and possible physical and chemical contributions that could be evaluated and controlled for, thereby providing high reliability and accuracy of electrochemical measurements of extracellular glucose fluctuations.

Briefly, glucose sensors are prepared from platinum-iridium wire (180 μm diameter), with a ~1-mm sensing cavity at the tip and a sensing area of ~0.56 mm^2^. The active electrode is incorporated with an integrated Ag/AgCl reference electrode. On the active surface of glucose sensors, glucose oxidase converts glucose to glucono-1, 5-lactone and H_2_O_2_, which is detected as an oxidation current at +0.6 V (Hu and Wilson, 1997; Wilson and Gifford, 2005). The contribution of ascorbate to the measured current is competitively reduced by co-localizing ascorbic acid oxidase on the active surface of the sensor to convert electroactive ascorbate to non-electroactive dehydroascorbate and water. A negatively charged Nafion polymer layer under the enzyme layer further helps to exclude endogenous anionic compounds. The currents from all sensors were passed to a computer via a potentiostat (Model 3104, Pinnacle Technology, Inc.), and all electrochemical data were sampled at 1 Hz using the PAL software utility (Version1.5.0, Pinnacle Technology, Inc.).

Immediately before and after each *in vivo* experiment, all sensors were calibrated *in vitro* in phosphate buffered saline (pH 7.3, t°=22–23°C) to determine their glucose sensitivity and selectivity against ascorbate. Since the current response to glucose directly depends upon temperature and this dependence is very stable across multiple *in vitro* tests for different substrate-sensitive sensors (Kiyatkin et al., 2013), all sensitivity values were corrected for 37°C (+96%).

Within the physiological range of glucose levels (Fellows and Boutelle, 1993; McNay and Gold, 1999), glucose sensors used in this study produced incremental linear current increases, with an averaged sensitivity 10.92 nA/1 mM (37°C). Glucose sensors showed low sensitivity to ascorbate (0.13 ± 0.05 nA/25 μM at 22–23°C) and, as showed previously, were only minimally sensitive to dopamine at its physiological levels (5–50 pA/10–100 nM). The mean post-recording calibration curve for glucose sensors was almost identical to the pre-recording curve, and sensitivity and selectivity remained virtually unchanged after 6–7 h of *in vivo* recording (10.05 nA/1 mM).

### Experimental Protocol

Electrochemical recordings were performed inside of an electrically-shielded Plexiglas chamber of a similar size that was used during training. A video camera recorded animal behavior, which was synchronized with electrochemical data during analysis. On the day of electrochemical recording, rats were minimally anesthetized with isoflurane and a calibrated glucose sensor was inserted into the NAc through the guide cannula. The rat was then placed in the testing chamber and the sensor was connected to the potentiostat via an electrically shielded cable and a multi-channel electrical swivel. Testing began a minimum of 120 min after sensor insertion to allow the baseline current to stabilize. First, to verify sensor functionality and the specificity of drinking-induced glucose responses, rats were exposed to a sensory stimulus that was not related to our behavioral task: a 1-min presentation of a novel object (a small glass beaker), that had some similar sensory properties to the glass bottle used in the drinking behavior. This control test was important for evaluating the contribution of sensory input and arousal to changes in glucose during drinking behavior. Fifteen min after the sensory control (and >150 min after the session start), rats were presented with a glass drinking tube identical to that used in training filled with 10% glucose solution that was secured to the chamber wall for 2–4 h. In addition to video recording, the change in consumed volume was recorded every 4 min from the moment of the tube presentation.

To distinguish the behavioral contributions to the NAc glucose response, we also examined changes in NAc glucose currents during passive injections of 10% glucose in three rats that were implanted with chronic intra-gastric catheters. At the start of these experiments, the catheter injection port was connected via an extension tube to a syringe filled with 10% glucose located outside the recording chamber. This permitted stress- and cue-free delivery of glucose. The volumes and durations of passive glucose infusions were similar to natural drinking bouts seen in behaving rats (4 ml or 400 mg and 8 ml or 800 mg, delivered over 4 and 8 min, respectively). Each of three rats received two passive injections: 1 h before and 1 h after their free-drinking session. As shown previously, similar volumes of directly injected water have no effect on NAc glucose (Wakabayashi et al., 2015).

### Histology

At the end of the recording, rats were removed from the cage, lightly anesthetized with isoflurane (<2 min), and the biosensor was removed for post-recording calibration. Then rats were deeply anesthetized and transcardially perfused with PBS followed by 10% formalin. Sensor placements were verified on 45 μm brain slices using the stereotaxic atlas of Paxinos and Watson (1998).

### Data Analysis

Our analysis paradigm was based on the behavior observed in experienced rats given unrestricted access to a 10% glucose solution. During the recording session, all rats exhibited a highly consistent pattern. When initially presented with the drinking tube, rats approached the tube and began drinking after a discrete latency. This drinking bout was sustained and continuous, followed by a much longer period of non-drinking. During this time the rat typically moved from the drinking tube to the opposite corner and transitioned to a quiet, sleep-like state. All rats within our sample then engaged in a second, well-defined bout of self-initiated drinking that was also followed by a second prolonged period of inactivity. Further consummatory behavior after these two bouts became more variable. Therefore, the dynamics of glucose were analyzed with respect to three critical events during the first bout (the initial tube presentation, start of drinking and end of drinking) and two events during the second bout (start and end of self-initiated drinking).

Since equally trained rats during an unrestricted behavioral task showed variable latencies to initiate drinking after the tube presentation and exhibited different durations of drinking, we first analyzed relative changes in glucose (as the reported oxidation current) preceding and following key behavioral events (peri-event analysis) with respect to the pre-event baseline (=0). These data were analyzed at two time scales. Slow time-course analyses (30-s or 1-min bins) were used to represent tonic changes in NAc glucose occurring within 30–60 min of the event of interest. For a more precise picture of rapid fluctuations in NAc glucose associated with the same event, we also analyzed the same data using 2- or 4-s bins within a shorter analysis window. Current data were transformed into estimated concentration values based on sensitivity of each sensor determined during *in vitro* calibrations and corrected for physiological temperatures (37°C). Data were also expressed as a percent change with respect to basal glucose levels. As shown previously, these basal levels of glucose in the NAc in awake, quietly resting rats are in the range of 540–700 μM (Kiyatkin and Lenoir, 2012; Wakabayashi and Kiyatkin, 2015; Wakabayashi et al., 2015). These values are close to previous electrochemical and microdialysis estimates conducted in awake animals (0.4–0.7 mM; Fellows et al., 1992; Lowry et al., 1998; McNay and Gold, 1999) but lower than data obtained in anesthetized rats (Silver and Erecińska, 1994).

One-way repeated-measures (RM) ANOVAs were used for statistical evaluation of changes in glucose currents preceding and following the events of interest. If a significant main effect was detected, individual data points were compared against the peri-event baseline point using Fisher *post hoc* tests to detect the latency and duration of this change.

The same analytical strategy was employed for analyzing changes in glucose levels induced by sensory stimuli and by passive intra-gastric glucose infusion. For text clarity, most in-depth statistical results are shown in figure captions.

## Results

Data were obtained in seven rats that consistently showed drinking behavior during the recording session, and had artifact-free electrochemical recordings. During the recording session, all rats rapidly initiated glucose drinking after the introduction of the drinking tube into the cage. The latency varied between 22 and 90 s, with a mean of 61.4 ± 10.2 s and median 59 s. The initial drinking bout in all rats was continuous, where they consumed 7.6 ± 1.7 ml of solution during 269.1 ± 41 s (4.5 ± 0.7 min). This period of initial continuous drinking was followed by a much larger time interval (range: 53–145 min; mean 81.8 ± 13.9 min) when rats did not drink and were typically in sleep-like state. Eventually, all rats resumed drinking but the duration of the second bout (mean: 187.3 ± 19.0 s, median 177 s) and the volume consumed (4.4 ± 0.78 ml) was less than for the first bout.

### Glucose Drinking Initiated by Tube Presentation

**Figure 1:**
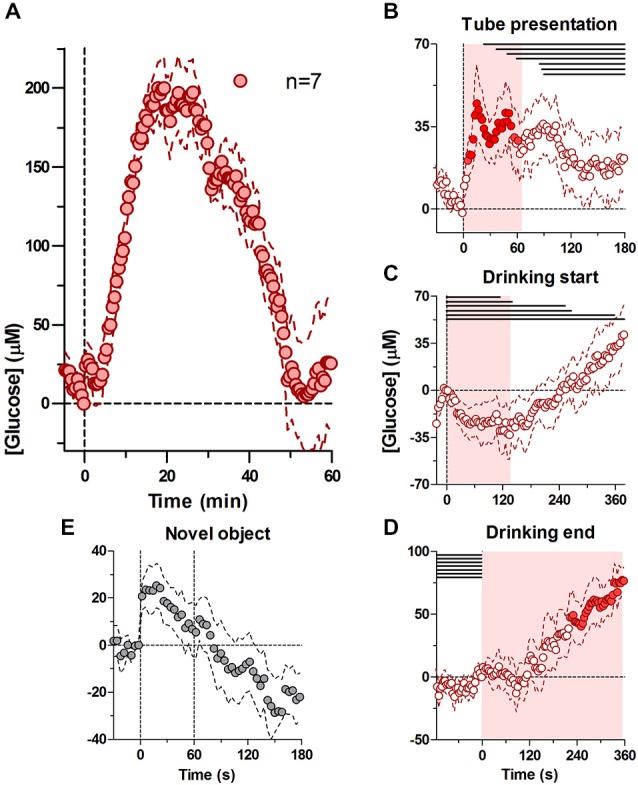
**Dynamic changes in nucleus accumbens (NAc) extracellular glucose during the cue-initiated drinking bout.** Panel **(A)** shows overall changes in glucose for 60 min after the cue-initated bout (30-s bins). **(B)** shows rapid changes in glucose (2-s bins) after the presentation of the drinking tube, while **(C,D)** show changes in glucose (4-s bins) at the start of drinking and end of drinking, respectively. There was a significant increase in [glucose] for 67 s after the tube presentation (**A**, *F*_(6,204)_ = 1.51, *p* < 0.05), prior to the onset of drinking (horizontal lines in **B**–**D**). At the onset of drinking, there was a significant decrease in glucose for 138 s (**C**, *F*_(6,210)_ = 1.51, *p* < 0.05), when the majority of rats were engaged in drinking. At the end of drinking, there was a significant increase in glucose levels (**D**, *F*_(6,540)_) = 13.94, *p* < 0.05). Shaded areas represent the duration of the main effect in (**B**–**D**), while filled symbols show individual points that were significant when compared to baseline using a Fisher test. **(E)** shows the NAc glucose response during 1-min presentation of a novel object.

Figure 1A shows relative changes in NAc glucose with respect to the moment of tube presentation and analyzed at a 30-s time resolution. At this timescale, glucose levels showed a two-component increase. First, glucose levels rose immediately and rapidly after the tube presentation, reaching a modest peak (20–30 μM or 4–5% above baseline) and returning to the baseline shortly thereafter (~3 min). A second increase began ~5 min after the tube presentation and after the cessation of drinking; it was much larger in magnitude (mean ~200 μM or 30% above baseline) and slower in dynamics (time to peak ~20 min, return to baseline ~50 min).

To determine the relationship between glucose dynamics and individual behavioral events we analyzed the same data using 2- and 4-s bins with respect to three events: (i) tube presentation; (ii) the start of drinking; and (iii) the end of drinking. As shown in Figure 1B, glucose levels rapidly increased within seconds after tube presentation (*F*_(6,204)_ = 1.51, *p* < 0.05). Importantly, this increase peaked at ~18 s (mean ~50 μM) before the onset of drinking in any of the rats and became progressively weaker as rats began drinking. Within this time scale, this increase was similar in magnitude and latency to the accumbal glucose response to the novel object presentation (Figure 1E). When the data were analyzed with respect to the onset of drinking (Figure 1C), glucose levels modestly (~25 μM) but significantly decreased during drinking compared to its immediate baseline at the drinking onset (*F*_(6,210)_ = 1.51, *p* < 0.05), which was elevated compared to pre-test levels (+39.45 ± 10.8 μM). This decrease began to slowly transition into a slow increase from ~120 s after the start of drinking, when some rats stopped drinking. When analyzed with respect to the end of drinking (Figure 1D), glucose levels remained unchanged for ~120 s but then began to increase strongly and persistently (*F*_(6,540)_ = 13.94, *p* < 0.05), creating a large post-consumption glucose elevation also seen with slow-scale analysis in Figure 1A.

### Self-Initiated Drinking

**Figure 2:**
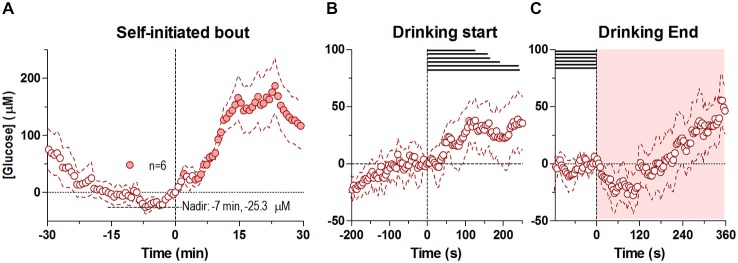
**Dynamic changes in NAc extracellular glucose during the self-initiated drinking bout.** Panel **(A)** shows overall changes for 30 min before and after the entire second, self-initiated bout (30-s bins), where there was a significant change in [glucose] for the entire analysis interval (*F*_(5,450)_ = 8.44, *p* < 0.05). Individual points significantly different from the [glucose] nadir (7 min before the onset of drinking, −25.3 μM), are shown as filled symbols. **(B,C)** show rapid changes in glucose (4-s bins) after the start and end of self-initiated drinking, respectively. While there was no significant change in [glucose] after the start of drinking, there was a significant increase overall after the end of drinking (*F*_(5,450)_ = 5.50, *p* < 0.05). No individual point in **(C)** was significantly different from baseline. Durations of drinking in each subject are shown as horizontal lines in **(B,C)**, shaded areas represent the duration of the main effect on each graph. Due to technical complications, one subject was removed from this analysis (*n* = 6).

In contrast to the first drinking bout, which began shortly after the drinking tube was introduced in the cage, the second bout was initiated spontaneously, without any environmental cues. Drinking in this case began when glucose levels were near its nadir from the preceding bout’s large increase (Figure 2A). Similar to the first bout, the second drinking bout was also associated with two unequal glucose peaks: a smaller one seen around the start of drinking and a larger post-consummatory one. Unlike the first bout, at the rapid timescale there was only a modest, non-significant increase in glucose at the start of drinking that persisted through the end of the behavior (Figure 2B). However, at the slower timescale, there was a steady increase in glucose levels from its pre-drinking nadir of ~ −25.3 μM that occurred ~7 min before the start of drinking, which became significant just after the onset of self-initiated drinking (−7 to +1 min, *F*_(5,60)_ = 2.30 *p* < 0.05). After the end of the self-initiated bout, glucose levels slightly decreased for less than ~120 s (Figure 2C), before beginning to increase robustly, mirroring the second robust increase seen after the first drinking bout.

### The Overall Dynamics of NAc Glucose Fluctuations Associated with Drinking Behavior

**Figure 3:**
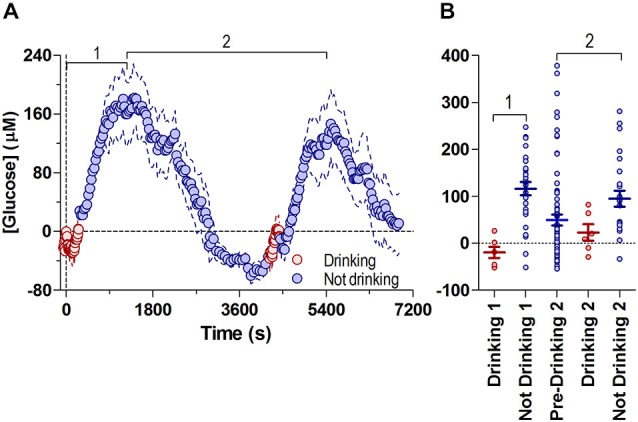
**Fluctuations in NAc [glucose] during both drinking bouts relative to the initial drinking bout’s baseline**. Panel **(A)** shows overall changes in glucose concentrations during periods of drinking (8-s bins) and no drinking (30-s bins), with respect to the first bout’s initial baseline (=0), and are combined into one common time-scale. Data shown for the median time periods for each behavior (pre-drinking 1: −59 s, duration of drinking 1: 266 s, time between end of drinking 1 and start of drinking 2: 4253 s duration of drinking 2: 177 s). **(B)** shows the glucose concentration from all individual subjects (4 min bins) during drinking and no drinking periods in **(A)**. Bout 1 is defined as the period from the onset of drinking 1 to the first peak in glucose concentration (1180 s), bout 2 is defined as the time between the first peak in glucose concentration 2, through the second drinking episode, to the peak in glucose concentration after the second bout (1210 s to 5494 s). Lines in **(B)** show mean ± SEM glucose concentration relative to the start of Drinking 1 (Drinking 1: −19.3 ± 11.74 μM, No Drinking 1: 116.5 ± 13.96 μM, Pre-Drinking 2: 49.69 ± 12.07 μM, Drinking 2: 23.07 ± 17.53 μM, No Drinking 2: 95.14 ± 17.17 μM).

Figure 3A shows the changes in NAc glucose associated with both drinking bouts on the same timescale, where both phasic and tonic changes are shown relative to the baseline before the start of the first drinking bout.

Changes in NAc glucose were highly cyclic, correlating with glucose-drinking behavior. Short episodes of drinking were associated with low NAc glucose levels near the pre-drinking baseline, while much longer periods of non-drinking were linked to a larger range of increased glucose levels. Consistent with the volumes consumed during each bout (7.6 and 4.4 ml), the post-drinking tonic increase was proportionally larger in its magnitude for the first vs. second bout (mean ~180 μM vs. ~147 μM or ~30 vs. 25% above baseline), suggesting that this rise in brain glucose is the result of ingested glucose reaching the NAc.

To examine the possible relationship between tonic, post-ingestion glucose increases and drinking behavior, we compared the relative change in glucose levels (every 4 min) in each rat while they were either drinking or not drinking, relative to the first bout’s pre-drinking baseline (Figure 3B). Sustained drinking only occurred when NAc glucose levels were near baseline levels, and drinking did not occur when glucose values were greater than ~90 μM. Therefore, these data suggest that large increases in brain glucose resulting from ingestion can negatively modulate drinking behavior. Likewise, falling levels of brain glucose to or below baseline levels appear to increase the probability of drinking.

### Passive Intra-Gastric Glucose Delivery

**Figure 4:**
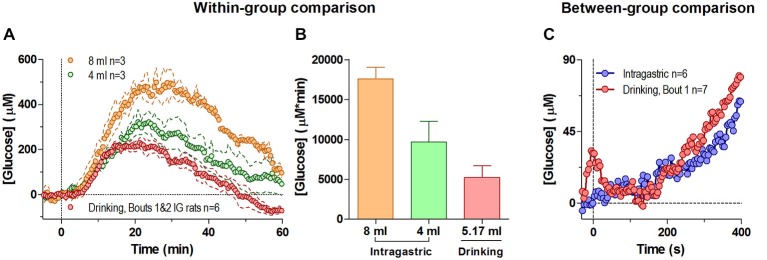
**Changes in NAc [glucose] induced by intra-gastric glucose injections**. Panel **(A)** shows overall changes in NAc glucose for 60 min after intra-gastric glucose injections (4 and 8 ml) and after drinking (mean 5.17 ml) in the same rats (30-s bins). **(B)** compares the duration and magnitude of the response (as area under the curve) for each condition in **(A)**, where there was an overall main effect (One-Way RM ANOVA, *F*_(2,4)_ = 9.45, *p* < 0.05), and the difference between 8 mg and drinking approached significance *p* = 0.051. **(C)** compares the initial response between all rats when they began drinking as a result of the tube presentation, and when a subset of rats received an intragastric injection of glucose (4 and 8 mg), relative to the pre-presentation and pre-injection baseline, respectively. During this time interval the response to a 4 and 8 ml intragastric injection was not significantly different, and was combined. For clarity, error bars not shown.

Passive administration of glucose via an intra-gastric catheter resulted in a large elevation of NAc glucose levels (Figure 4A). While the latency and the initial components of the response were similar for both doses (Figure 4A, 4 and 8 ml or 400 and 800 mg), the amplitude and duration of the response expressed as the area under the curve was larger for the large-dose injection (Figure 4B; *F*_(2,4)_ = 9.45, *p* < 0.05). Importantly, the increase induced by passive glucose injection was qualitatively larger than that occurring after glucose drinking despite a larger volume of consumed glucose. Although the second, slower increase in glucose levels were generally similar within the first 5 min regardless of the dose or route of delivery, we found clear differences in glucose dynamics between tube presentation-mediated consumption and passive glucose delivery within the first 60 s of analysis (Figure 4C). Within this short window, in behaving rats glucose levels phasically increased relative to pre-test baseline before drinking began (i.e., after the tube presentation) and rapidly decreased at the onset of drinking. Comparatively, these changes are noticeably absent in rats receiving a similar volume of glucose intra-gastrically. This relatively small but unique difference may represent the functional contribution of the NAc towards initiating consummatory behavior under stimulus control.

## Discussion

The present study produced several findings. First, we show that unrestricted glucose-drinking behavior results in a relatively large and prolonged elevation in extracellular levels of glucose in the NAc (150–300 μM or 25–30% above baseline). Second, we demonstrate that this change has two temporally distinct components: a highly phasic but relatively small behavior-related rise and much more powerful tonic elevation that results from the arrival of consumed glucose into brain tissue. Third, we found that large post-ingestion increases in NAc glucose (>100 μM) are consistently associated with periods of no drinking, suggesting that the central action of ingested glucose inhibits drinking behavior by inducing a prolonged pause in activity consistently seen between repeated drinking bouts. Finally, the difference in NAc glucose responses found between active, behavior-guided and passive glucose delivery confirms that motivated behavior is associated with glucose metabolic use.

### Two Components of Glucose Entry in Brain Tissue and their Mechanisms

Brain activity is highly dependent on glucose delivery, which comes entirely from the arterial blood via a concentration gradient-dependent, facilitated diffusion via the GLUT-1 transporter (Fellows and Boutelle, 1993; Duelli and Kuschinsky, 2001; Barros et al., 2005). Consistent with previous studies, our present data suggest two temporally distinct mechanisms underlying glucose entry in brain tissue. First, the large NAc glucose elevation evident after both active drinking and passive intra-gastric glucose delivery results from its gradient-dependent entry due to a rise in blood levels and increasing difference between the concentration in blood and brain. This slower, tonic glucose rise has discrete, minute-scale onset latencies and is relatively large in magnitude (200–400 μM or 40–70% above baseline for amounts of glucose typically consumed in one bout by trained rats), and slowly disappears within 40–60 min. At this concentration range, glucose can unambiguously interact with neuronal and glial glucoreceptors on multiple central neurons (Levin et al., 2002; Burdakov et al., 2005; Karnani and Burdakov, 2011), thus inducing neural effects.

In addition to the large post-ingestion rise, NAc glucose levels phasically fluctuated in association with some critical behavioral events. While these phasic fluctuations were much smaller (~50 μM, or within 10% of baseline), they were exceptionally phasic, occurring in the scale of seconds. These rapid glucose fluctuations are likely a reflection of “neurovascular coupling” (Attwell et al., 2010; Mergenthaler et al., 2013), that is triggered by local changes in neuronal activity (Kiyatkin and Lenoir, 2012), leading to the vasodilation of proximal blood vessels, and resulting in enhanced glucose entry into brain tissue.

### Phasic, Behavior-Related Components of the NAc Glucose Response

When presented with the drinking tube, NAc glucose levels rapidly rose and peaked (~50 μM) before the rats began drinking (Figure 1B). While this initial, tube presentation-related increase could indicate rapid glucose entry into the extracellular space in response to a reward-related cue that triggers seeking, approach to the tube, and finally drinking, a similarly rapid glucose rise occurred in response to a novel arousing stimulus not directly related to our behavioral task (Figure 1E).

The similarity in NAc glucose responses between two stimuli with different motivational values suggests that rapid glucose entry in the NAc extracellular space could be a manifestation of generalized arousal, a non-specific neural activation that is an essential pre-condition for any behavior. Accumbal neurons are typically phasically excited in response to sensory stimuli of different modality (Carelli and West, 1991; Schneider, 1991; Kiyatkin and Rebec, 1996; Rebec, 1998; Kiyatkin and Brown, 2007), including reward-predicting cues (e.g., Nicola et al., 2004a,b). However, in our task arousal initiated by the tube presentation was followed by the start of drinking, a discrete event immediately preceded by exploratory and approach behavior, that can also increase NAc neuronal activity (Taha and Fields, 2005; Taha et al., 2007). When analyzed with respect to the onset of drinking, glucose levels also appeared to be near their maximal point (see Figure 1C), suggesting that neural activity triggered by a reward-predicting cue remains elevated until the rat started drinking.

Furthermore, cessation of this neural activation could explain a rapid, transient but relatively modest decrease in glucose levels (~25 μM) that occurred during the first, tube-initiated drinking bout. The activity of accumbal neurons is known to decrease during drinking (Nicola et al., 2004a,b; Roitman et al., 2005; Taha and Fields, 2005; Krause et al., 2010) and decreases in neuronal activity have been suggested in fMRI studies in humans (Smeets et al., 2007; Page et al., 2013). While the cessation of previous neural activation could explain this transient, relative decrease in brain glucose during glucose drinking, it is difficult to speculate on further neuronal changes due to the post-ingestion glucose entry that results in robust elevation in NAc glucose levels.

While direct neural signaling to vessels due to neural activation is critical for functional hyperemia and a rapid rise in extracellular glucose (Attwell et al., 2010), recent data suggest the involvement of other neural substrates, particularly astrocytes, and various signaling molecules in regulating the local vascular response (Iadecola and Nedergaaard, 2007; Attwell et al., 2010; Howarth, 2014). However, this vascular response is initiated via a neural mechanism and fast signaling to local blood vessels appears to be the trigger for functional hyperemia and a rapid rise in extracellular glucose. The causal relations between neural activation and glucose entry into brain tissue have been previously substantiated for the NAc by using local microinjections of glutamate nearby the tip of glucose sensors (Kiyatkin and Lenoir, 2012). Local excitation of accumbal cells in this case resulted in a rapid, dose-dependent rise of extracellular glucose comparable to or higher than that seen under behavioral conditions.

In contrast to tube-initiated drinking, the NAc glucose dynamics differed with the self-initiated second bout, which was shorter in duration and resulted in less glucose consumption (Figure 2). In this case, there was a conspicuous lack of a robust phasic response prior to the onset of drinking (Figure 2B), possibly due to lack of a discrete stimulus. Rather, there was a steady, albeit slower increase in glucose levels from its nadir for minutes preceding the self-initiated bout (Figure 2A), a period when the rat awakened, increased its activity, and exhibited exploratory behavior. This increase continued throughout the second bout, possibly reflecting modest but sustained neural activation related to exploratory, approach and consummatory behavior that is normally masked by the more robust phasic glucose changes during cue-initiated drinking. This slower increase ceased at the end of drinking (Figure 2C) before rising again due to glucose entry from the stomach. Hence, these data suggest that the metabolic demand of the NAc, and by extension its activity is less prominent during self-initiated drinking, than when the behavior is stimulus initiated.

Taken together, it is possible that the NAc permissively requires elevated glucose to properly process arousing stimuli of different nature in order to effectively evaluate the appropriate behavioral response to a selected few (Nicola, 2007). Regardless, from a metabolic point of view, glucose rapidly enters NAc brain tissue to provide necessary energetic resources for its future use.

### Post-Consumption Increase in Brain Glucose and its Possible Functional Relevance

While often either considered as self-evident or not considered at all, our study showed conclusively that natural unrestricted glucose drinking in non-deprived but highly trained rats results in a relatively large elevation in NAc glucose (150–350 μM), well above the change that are able to affect multiple glucoreceptors expressed on brain neurons, primarily in the hypothalamus and brain stem (Burdakov et al., 2005; Routh, 2002; Levin et al., 2011). A similar, post-ingestion tonic glucose rise has been previously reported after a fixed-volume drinking (5 mL) initiated by cup presentation (Wakabayashi et al., 2015). This similarity precludes the suggestion that the post-ingestion increase is related to the forced cessation of drinking of a limited quantity, and is not related to brain activation due to increased post-drinking searching or a neural correlate of “frustration”. Thus, in addition to its well-known role as a metabolic substrate, ingested glucose reaches the brain at a behaviorally relevant timescale in significant amounts to produce direct effects on CNS neurons that are involved in regulating multiple neural functions. Among these targets, interaction of glucose with orexin/hypocretin hypothalamic neurons could be especially important because increases in brain glucose levels strongly inhibit the activity of these “arousal-related” cells (Venner et al., 2011), possibly determining inhibition of further drinking, and the development of a sleep-like, satiety state. On the other hand, this inhibitory effect on behavior ceases when brain glucose levels return to baseline and the rat increases their activity levels resulting in another drinking bout (Figure 3A). Therefore, large, tonic, post-ingestion elevations in brain glucose appear to play an important role in regulating glucose-drinking behavior in well-trained, unrestricted rats. These elevations are cyclic and are tightly associated with behavior, where relatively short drinking bouts (2–4 min) are alternated with much longer periods of no drinking and sleep-like activity (40–70 min; Figure 3B). Of course, in light of these data further studies are necessary to establish a causal relationship between brain glucose levels and drinking behavior.

By using intra-gastric glucose delivery, we confirmed that the large glucose elevation occurring in behaving rats at the end of the drinking bout has a peripheral, exogenic source. This component of the NAc glucose response is dynamically distinct from the much smaller, phasic glucose fluctuations that result from accelerated entry of endogenous glucose due to local neural activation and subsequent increases in proximal blood flow. Interestingly, the NAc glucose response after passive glucose delivery was qualitatively larger in terms of magnitude and duration than the response occurring during glucose delivery via active behavior. This difference could be explained by the behavior-associated metabolic consumption of glucose that is absent when glucose is passively delivered. While indirectly confirming that motivated behavior is accompanied by glucose consumption by brain cells for their metabolic needs, it remains challenging to quantify dynamically this effect via this approach.

Interestingly, the tonic glucose rise induced by intra-gastric glucose delivery in this study was quantitatively weaker than that occurred in rats with moderate training and limited access to glucose (Wakabayashi et al., 2015). While this finding could suggest the development of tolerance in brain glucose response in animals with extensive glucose experience, it should be noted that this difference could be also explained by the older age and respectively much larger weights of rats used in this study. Further studies are clearly necessary to verify this finding.

### Conclusions and Functional Implications

While slow changes in brain glucose could be detected by microdialysis (McNay and Gold, 1999; Dunn-Meynell et al., 2009; Krebs-Kraft et al., 2009), enzyme-based glucose biosensors coupled with high-speed amperometry allowed us to reveal rapid, behavior-associated glucose fluctuations that occur in the scale of seconds and temporally resolve them from slow changes occurring in the scale of minutes. These behavior-associated fluctuations are relatively small in magnitude (~5–10% of baseline), but highly dynamic, possibly reflecting rapid changes in neuronal activity and subsequent changes in local blood flow. Therefore, the changes in neuronal activity are not only critical for the organization and regulation of motivated behavior, they appear to be essential for providing necessary energetic resources (i.e., glucose and oxygen) for maintaining this behavior. Rapid detection also clearly revealed large, post-ingestion elevations in brain glucose levels that occur at a behaviorally relevant timescale, could affect neuronal activity, and are tightly correlated with behavioral activity states. These large changes appear to be involved in the regulation of drinking behavior, determining its cyclicity (periodicity) and highlighting the importance of the direct action of glucose on the brain.

## Conflict of Interest Statement

The authors declare that the research was conducted in the absence of any commercial or financial relationships that could be construed as a potential conflict of interest.
